# Clopidogrel, a P2Y12 Receptor Antagonist, Potentiates the Inflammatory Response in a Rat Model of Peptidoglycan Polysaccharide-Induced Arthritis

**DOI:** 10.1371/journal.pone.0026035

**Published:** 2011-10-18

**Authors:** Analia E. Garcia, Sripal R. Mada, Mario C. Rico, Raul A. Dela Cadena, Satya P. Kunapuli

**Affiliations:** 1 Sol Sherry Thrombosis Research Center, Temple University School of Medicine, Temple University Hospital, Philadelphia, Pennsylvania, United States of America; 2 Department of Physiology, Temple University School of Medicine, Temple University Hospital, Philadelphia, Pennsylvania, United States of America; 3 Department of Pharmacology, Temple University School of Medicine, Temple University Hospital, Philadelphia, Pennsylvania, United States of America; University of Leuven, Rega Institute, Belgium

## Abstract

The P2Y12 receptor plays a crucial role in the regulation of platelet activation by several agonists, which is irreversibly antagonized by the active metabolite of clopidogrel, a widely used anti-thrombotic drug. In this study, we investigated whether reduction of platelet reactivity leads to reduced inflammatory responses using a rat model of erosive arthritis. We evaluated the effect of clopidogrel on inflammation in Lewis rats in a peptidoglycan polysaccharide (PG-PS)-induced arthritis model with four groups of rats: 1) untreated, 2) clopidogrel-treated, 3) PG-PS-induced, and 4) PG-PS-induced and clopidogrel-treated. There were significant differences between the PG-PS+clopidogrel group when compared to the PG-PS group including: increased joint diameter and clinical manifestations of inflammation, elevated plasma levels of pro-inflammatory cytokines (IL-1 beta, interferon (IFN) gamma, and IL-6), an elevated neutrophil blood count and an increased circulating platelet count. Plasma levels of IL-10 were significantly lower in the PG-PS+clopidogrel group compared to the PG-PS group. Plasma levels of platelet factor 4 (PF4) were elevated in both the PG-PS and the PG-PS+clopidogrel groups, however PF4 levels showed no difference upon clopidogrel treatment, suggesting that the pro- inflammatory effect of clopidogrel may be due to its action on cells other than platelets. Histology indicated an increase in leukocyte infiltration at the inflammatory area of the joint, increased pannus formation, blood vessel proliferation, subsynovial fibrosis and cartilage erosion upon treatment with clopidogrel in PG-PS-induced arthritis animals. In summary, animals treated with clopidogrel showed a pro-inflammatory effect in the PG-PS-induced arthritis animal model, which might not be mediated by platelets. Elucidation of the mechanism of clopidogrel-induced cell responses is important to understand the role of the P2Y12 receptor in inflammation.

## Introduction

The P2Y12 receptor is essential for ADP-induced platelet aggregation [1], [2], [3] and in thrombus growth and stability [4]. Due to its important role not only in ADP-induced but also in other agonist-induced platelet functional responses, the P2Y12 receptor has become a successful target for anti-thrombotic drugs. The thienopyridine compounds such as clopidogrel and prasugrel are first converted to an active metabolite in the liver and the active metabolite irreversibly inactivates the P2Y12 receptor [5]. *Ex vivo* inhibition of the P2Y12 receptor by clopidogrel administration diminishes the rapid exposure of Tissue Factor, suggesting a role for the P2Y12 receptor in the pro-coagulant activity of platelets [6].

Antagonism of the P2Y12 receptor diminishes the extent of release from platelets of both the alpha and dense granules. The dense granules contain ADP and ATP which act on platelets and other bloods cells upon release [7]. Extracellular ATP has been shown to trigger mobilization of intracellular calcium stores in freshly isolated human neutrophils and monocytes, which results in either direct stimulation of some inflammatory responses or enhanced responsiveness to agonists [8]. The alpha granules contain several growth factors as well as chemokines that stimulate peripheral blood leukocytes [9], [10], [11], [12]. In addition, the P2Y12 receptor has been shown to potentiate arachidonic acid liberation, which can be converted to thromboxane A2 in platelets and to leukotrienes in peripheral blood leukocytes [13]. The leukotrienes thus generated play an important role in the inflammatory responses. Hence, platelet activation leading to release of granule contents and arachidonic acid liberation has been thought to contribute to inflammatory responses.

Rheumatoid arthritis (RA) is one of the most prevalent inflammatory diseases afflicting humans and several animal models are available that mimic the erosive arthritis in this pathological condition [14]. One such animal model applicable to the study of joint inflammation is the induction of erosive arthritis by peptidoglycan-polysaccharide (PG-PS) in susceptible animals [14], [15], [16]. PG-PS is a purified form of a polymer extracted from Group A *Streptococcus* cell walls [14]. When PG-PS is injected intraperitoneally *(i.p.)* in arthritis-susceptible species such as Lewis rat, it induces a chronic, erosive, and recurrent poly-arthritis, known to resemble human rheumatoid arthritis in clinical, histological and radiological detail [16], [17]. The primary immunogenic moiety of the PG-PS is the peptidoglycan. Arthritis develops as early as two days after PG-PS administration for a period of three to five days (acute phase), mediated by the complement system [18]. The arthritis-induced animal shows a remission period between 4 to 10 days followed by spontaneous reactivation of the joint inflammation that last for several weeks (chronic phase). This phase might be mediated by T cells proliferation and infiltration. In human RA there is evidence for platelet activation [19], which in turn may lead to neutrophil stimulation [20], [21], [22]. The PG-PS model thus represents an ideal experimental animal model to test potential drug interventions targeting platelets.

Antagonizing the P2Y12 receptors on platelets reduces the release of the granule contents [23]. Several anti-thrombotic drugs, such as clopidogrel, can act on the P2Y12 receptor thereby reducing platelet activation and granule release [24], [25]. Activated platelets release pro-inflammatory cytokines and nucleotides from granules at the site of vascular injury and as a result may trigger inflammation and thrombosis [26]. In addition, activated platelets release a number of chemokines from alpha granules that activate leukocytes [11]. Boilard *et al.* [19] identified micro-particles generated by activated platelets in joint fluid from RA patients. Although clopidogrel is a potent anti-thrombotic agent acting through the P2Y12 receptor, the effects of P2Y12 receptor antagonists have not been fully evaluated on chronic inflammatory responses. Therefore, in this study, we used clopidogrel in the PG-PS-induced arthritis model to evaluate its effects on inflammation.

## Results

### Clopidogrel increases the clinical and pathological manifestations of PG-PS-induced arthritis

**Figure 1:**
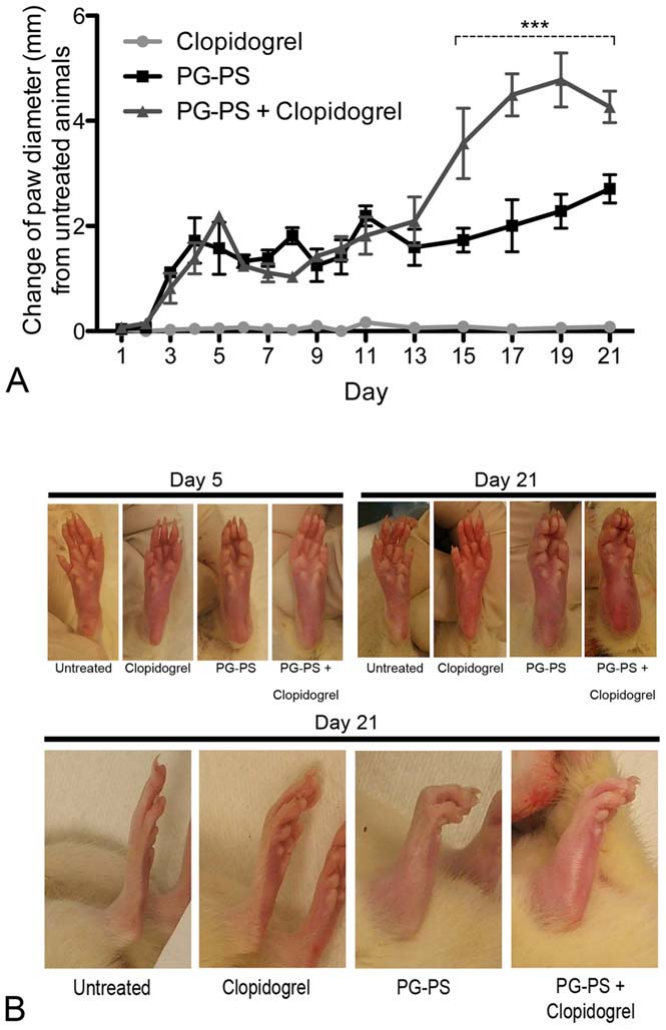
Effect of clopidogrel on the PG-PS-induced arthritis animal model. (**A**) Measurements of ankle joint diameter. Values represent the change of paw diameter in millimeters. Changes of paw diameter observed in the PG-PS+clopidogrel group were significantly increased when compare to the PG-PS group from day 15 to day 21, ***p<0.0001. (**B**) Photographic images of paw and ankle joints of the animals on day 5 and day 21.

Untreated animals and those treated with clopidogrel alone showed no changes in joint diameters during the course of the study. The effect of clopidogrel in this last group was tested during the course of the experimental protocol to assess a non-response pattern of platelets in response to ADP (data not shown). PG-PS-induced arthritic animals treated and untreated with clopidogrel showed an increase in the ankle joint diameter during both the acute and chronic phases. The increased joint diameter in PG-PS-induced arthritic animals was exacerbated in the group combining PG-PS with clopidogrel when compared with PG-PS alone and this joint diameter increase was significant during days 15 to 21 (***p<0.0001) (Figure 1).

**Figure 2:**
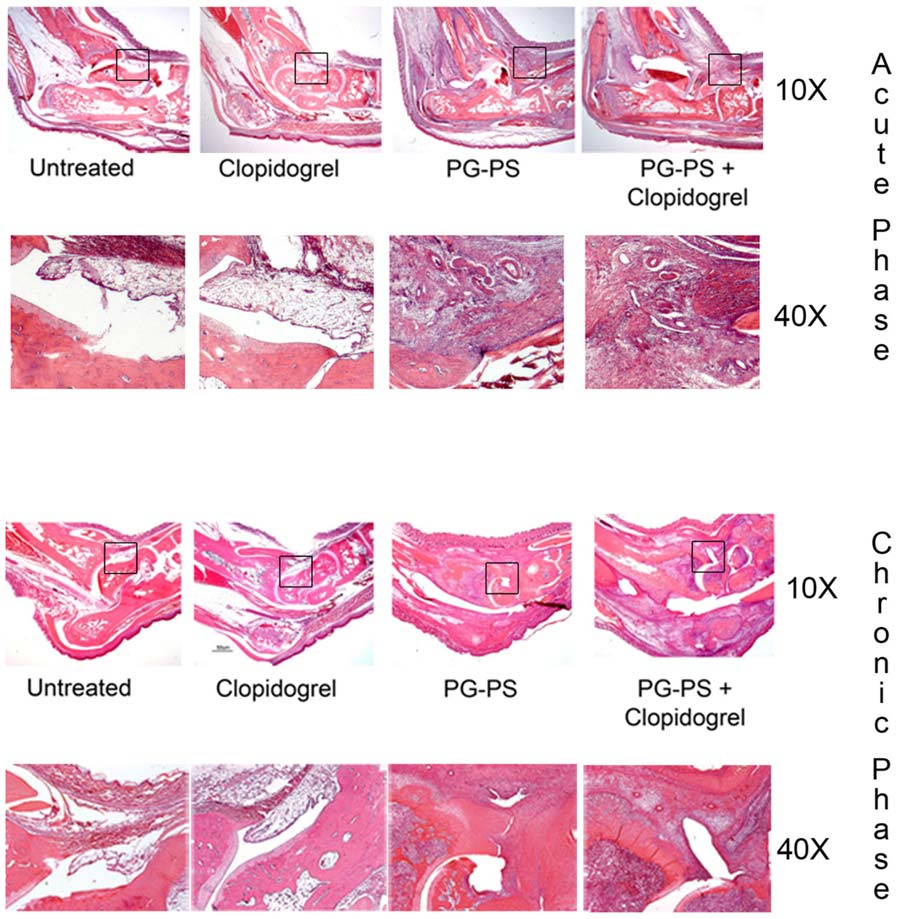
Histology of the ankle joint of the animals. 10× and 40× magnification of paraffin-embedded ankle sections stained with H&E, for the acute phase (day 5) and the chronic phase (day 21). The 40× magnification on day 5 shows leukocyte infiltration in the PG-PS-induced arthritis animals. Slides from the chronic phase in the PG-PS-induced arthritis animals showed fibrosis, pannus formation and severe leukocyte infiltration with more pronounced effects observed in the clopidogrel-treated animals.

**Figure 3:**
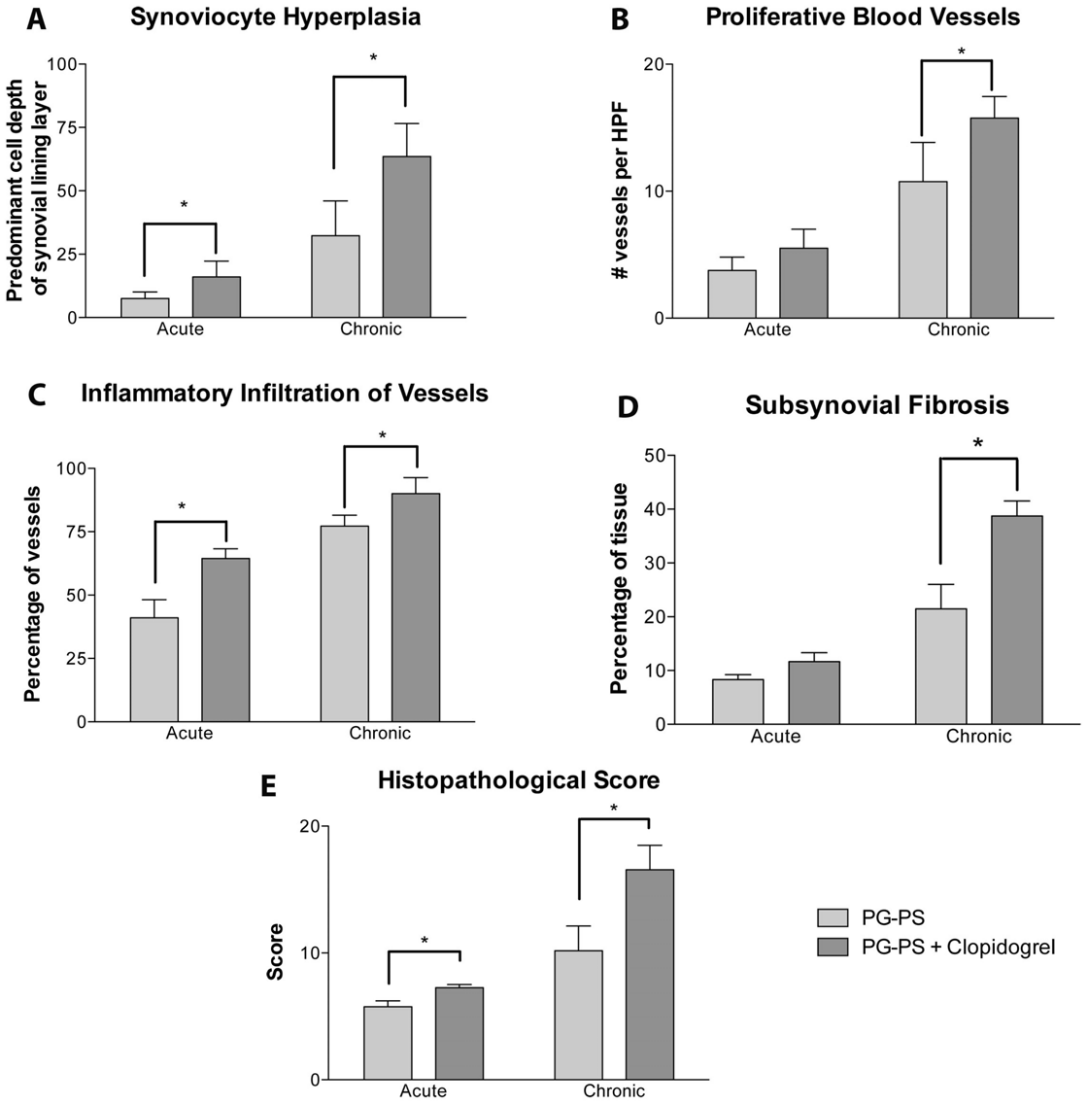
Four pathological parameters and the histopathological score from the histological sections of the acute and chronic phases. Synoviocyte hyperplasia (**A**), proliferative blood vessels (**B**), inflammatory infiltrates (**C**) sub-synovial fibrosis (**D**) and the histopathological score (**E**). Values plotted are the mean of the measurements ± SEM, (n = 3 per group) *p<0.05, PG-PS-treated with clopidogrel group vs. PG-PS group. Representation of the animals from the untreated and clopidogrel-treated groups was excluded to simplify graphs since these animals showed no inflammatory changes.

Histopathological changes were also seen between the groups. Untreated animals and those dosed with clopidogrel alone did not show any inflammatory changes. However, in PG-PS-treated animals there was a diffuse infiltration of inflammatory cells with severe synovial thickening, pannus formation, blood vessel proliferation, subsynovial fibrosis, subchondral inflammation and cartilage erosion. PG-PS-treated animals alone or in combination with clopidogrel exhibited a greater change in the joint inflammatory process. These changes observed were more pronounced in the PG-PS in combination with clopidogrel when compared with PG-PS alone (Figure 2). Inflammatory changes were measured and pathological scores were assigned as explained in detail in Materials and Methods. Scores for synoviocyte hyperplasia (in both acute and chronic phases), blood vessel proliferation (chronic), inflammatory infiltration (acute and chronic) and fibrosis (chronic) were increased in the PG-PS-treated animals in combination with clopidogrel when compared to the PG-PS-treated animals alone, *p<0.05 (Figure 3, panels A–D). Hind paw histopathological scoring system was used to assess the inflammatory responses, as reported previously [27]. A score of zero indicates a lack of inflammation, which was observed in both untreated animals and those dosed with clopidogrel alone. PG-PS-treated animals had an average score of 5.75±0.96 at day 5 rising up to 10.17±4.79 at day 21 (vs. untreated and clopidogrel-treated groups, *p*<0.05). PG-PS-treated animals in combination with clopidogrel demonstrated a significant increase in the histopathological score average, up to 7.25±0.5 at day 5 rising up to 16.56±5.81 at day 21 (vs. PG-PS-treated group *p*<0.05 for both day 5 and day 21, Figure 3, Panel E.) In summary, the data indicate that PG-PS administered in combination with clopidogrel is associated with marked inflammatory changes when compared with PG-PS alone.

### Clopidogrel administration in combination with PG-PS is associated with thrombocytosis, neutrophilia and leukocytosis

**Figure 4:**
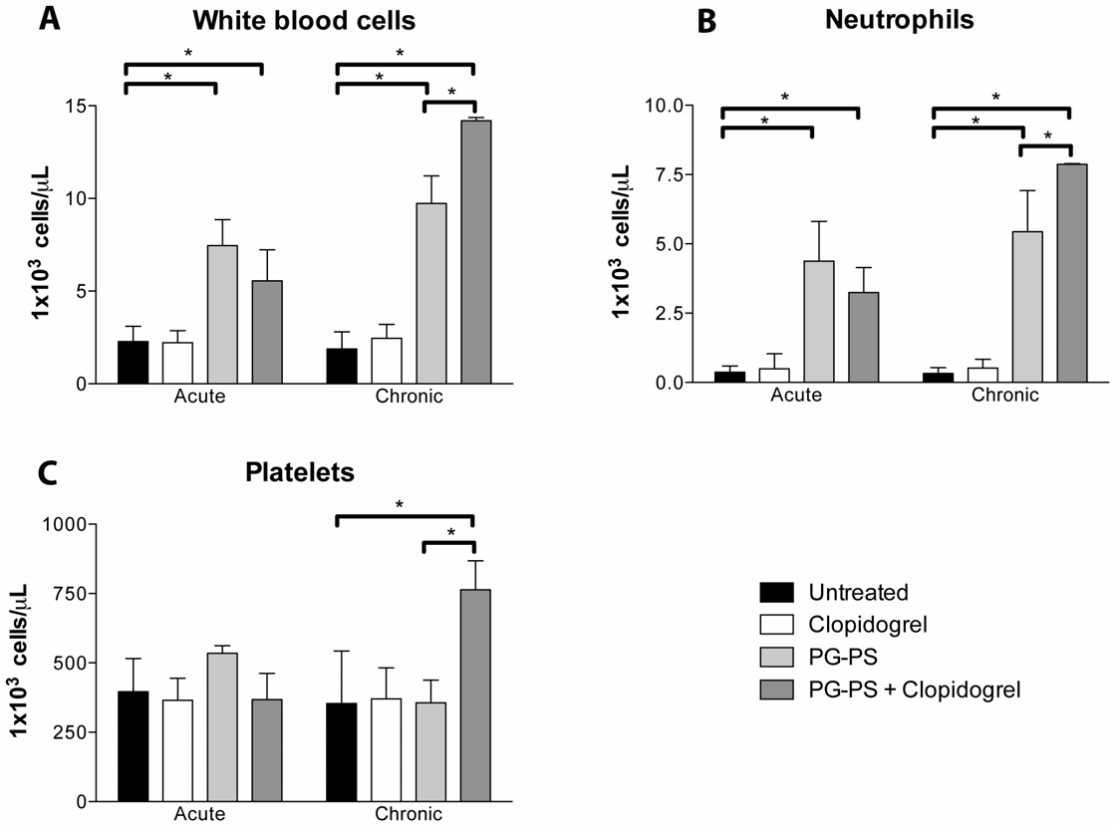
Blood cell counts of rats following clopidogrel treatment in PG-PS- induced arthritis model. Leukocyte (**A**), neutrophil (**B**), and platelet (**C**) cell blood counts at day 5 and day 21. All cell blood counts are plotted at 10^3^/µL. Values are mean ± SEM, (n = 6), untreated group (black bar), clopidogrel group (white bar), PG-PS group (light gray bar) and PG-PS-treated with clopidogrel group (dark gray bar), * *p*<0.05.

Upon completion of the two phases of the study, hematological parameters were evaluated in each group. There were no differences in the hematological parameters in clopidogrel-treated animals compared to untreated animals. In contrast, significant increases in total circulating leukocytes and neutrophils were observed in PG-PS-treated animals as well as PG-PS-treated animals in combination with clopidogrel, compared to untreated animals (*p*<0.05, Figure 4). Interestingly, platelet numbers were not significantly different in PG-PS-treated animals compared to untreated animals, but they increased significantly in PG-PS plus clopidogrel-treated animals in the chronic phase. Liver and kidney function was evaluated in all groups and did not account for the hematological changes described above (Table 1).

**Table 1:** Complete chemistry profile of experimental animals.

	Untreated	Clopidogrel	PG-PS	PG-PS+Clopidogrel
**CHOLESTEROL** (mg/dL)	83.5±7.54	70±14.85	83.25±13.67	87.67±14.57
**TRIGLYCERIDES** (mg/dL)	69.5±5.97	47.25±14.1	64.75±19.96	60.67±17.1
**ALT** (u/L)	86.75±50.92	56±10.17	55±4.08	46.67±4.16
**AST** (u/L)	189.75±80.51	117.50±53.57	94±30.6	117.34±36.96
**ALK** (mg/dL)	233.75±54.87	202±38.01	240.75±26.6	261±62.23
**TOTAL BILLIRUBIN** (mg/dL)	0.25±0.06	0.18±0.1	0.25±0.1	0.13±0.06
**TOTAL PROTEIN** (g/dL)	5.38±0.45	4.7±1.07	5.93±0.57	6.33±0.57
**ALBUMIN** (g/dL)	3.05±0.35	2.2±1.04	2.75±0.37	2.77±0.35
**GLUCOSE** (mg/dL)	193.25±36.44	211.5±36.97	195.25±33.33	182±11
**BUN** (mg/dL)	18.75±4.57	20.5±5.97	19±3.16	17±1.73
**CREATININE** (mg/dL)	0.33±0.22	0.43±0.1	0.43±0.05	0.37±0.06
**SODIUM** (mEq/dL)	142.2±2.26	141.50±2.01	142±0.57	141.5±2.22
**POTASSIUM** (mEq/dL)	4.58±1.05	4.67±0.97	4.59±0.09	5.48±0.9
**CHLORIDE** (mEq/dL)	105.3±0.57	104.8±1.02	104.95±2.05	104.2±1.02
**CALCIUM** (mg/dL)	10.35±0.37	9.7±1.36	10.28±0.52	10.83±0.38
**PHOSPHATE** (mg/dL)	5.08±0.25	4.93±0.93	5.73±0.83	6.43±0.85

Serum chemistry of rats following clopidogrel treatment in PG-PS-induced arthritis model (day 21). Values are mean ± SD, (n = 6). There are no statistical differences among groups.

### Clopidogrel administration in combination with PG-PS alters the plasma cytokine profile

**Figure 5:**
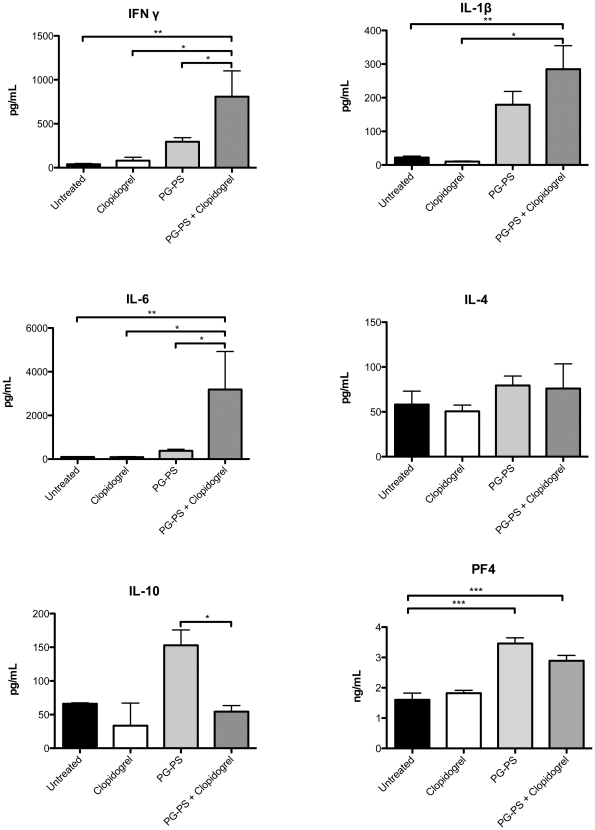
Plasma cytokine levels in rats following clopidogrel administration in PG-PS-induced arthritis model (day 21). Plasma IFN-γ, IL-β and IL-6 levels were elevated in the PG-PS-induced arthritis-treated clopidogrel group. In contrast, plasma levels of IL-10 were significantly lower in the PG-PS-induced arthritis rats compare to the PG-PS-induced arthritis animals alone. Plasma PF4 levels rose in PG-PS-induced arthritis animals (alone and with treatment with clopidogrel; however, no significant differences were observed between these two groups). There were no changes between groups in IL-4 plasma levels. Values represent the mean ± SEM, (n = 4), untreated group (black bar), clopidogrel group (white bar), PG-PS group (light gray bar) and PG-PS treated with clopidogrel group (dark gray bar), * *p*<0.05 PG-PS-induced arthritis group vs. PG-PS-induced arthritis treated with clopidogrel group.

Inflammatory responses are regulated by both pro-inflammatory and anti-inflammatory cytokines [28]. Thus, plasma levels of pro-inflammatory and anti-inflammatory cytokines in plasma were evaluated during the chronic phase of the study, namely IL-1β, IL-4, IL-6, IL-10, and IFN-γ. Baseline levels of these cytokines were unchanged in the untreated group or in the group treated with clopidogrel alone. However, significant changes were observed in the plasma levels of pro-inflammatory cytokines IFN-γ, IL-6, and IL-1β, in the PG-PS-treated animals, when compared with untreated animals or animals that were treated with clopidogrel alone. Furthermore, the pro-inflammatory cytokine profile was augmented in the PG-PS-treated animals in combination with clopidogrel when compared to PG-PS-treated alone (Figure 5). Notably, the anticipated increased in the anti-inflammatory cytokine IL-10 in response to PG-PS was blunted down to baseline values in the PG-PS-treated animals in combination with clopidogrel. Clopidogrel treatment alone was not responsible for such changes since the baseline values when compared with untreated group were not statistically significant (Figure 5). The levels of IL-4, an anti-angiogenic cytokine, were not changed in any of the four groups tested. PF4 plasma levels were increased in all PG-PS-induced arthritis animals when compared to untreated and clopidogrel-treated animals. However, treatment with clopidogrel in induced arthritis animals did not differ from the induced arthritis control animals.

**Figure 6:**
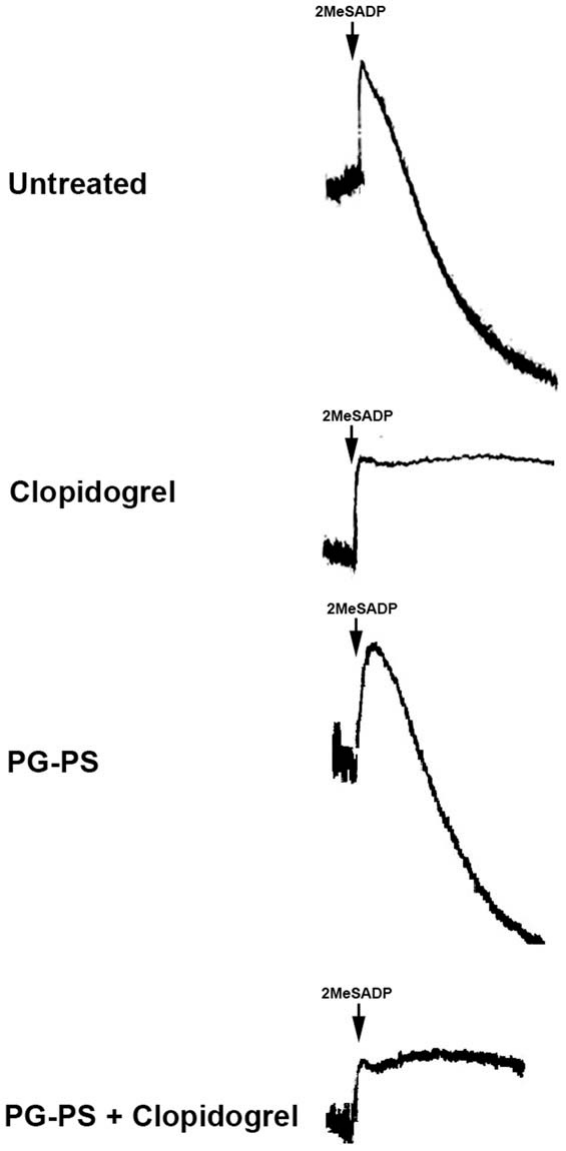
Platelet aggregation studies using 2MeSADP in PG-PS-induced arthritis model. Effect of 100 nM of 2MeSADP on platelet aggregation in isolated platelets from untreated animals, clopidogrel-treated animals, PG-PS-induced arthritis animals and PG-PS-induced arthritis+clopidogrel-treated animals. Traces are representative of three independent experiments.

To evaluate whether the use of PG-PS changed the platelet response to clopidogrel, platelet aggregation studies were performed in washed platelets from the four groups of animals. Platelets from the PG-PS-treated animals stimulated with 2MeSADP, a P2Y12 agonist, showed normal aggregation and secretion responses, compared to untreated animals. Platelets from the PG-PS-treated and clopidogrel-dosed animals showed diminished aggregation responses compared to those dosed with clopidogrel alone (Figure 6). Hence, PG-PS does not alter platelet reactivity to P2Y12 agonists nor affect clopidogrel activity on platelets.

## Discussion

We investigated the effect of clopidogrel on a rat model of erosive arthritis. Treatment with clopidogrel in animals with PG-PS-induced arthritis led to a significant increase in joint diameter and inflammation compared to animals with PG-PS-induced arthritis alone. There were no inflammation or joint diameter changes in animals treated with clopidogrel, indicating that clopidogrel alone does not have any effect in the joints of normal animals. However, clopidogrel in the presence of inflammation aggravated the course of the disease. Additionally, the clinical manifestations observed in the PG-PS-induced arthritic animals treated with clopidogrel also demonstrated leukocytosis and thrombocytosis.

The active metabolite of clopidogrel is normally metabolized in the liver [5]. Since PG-PS has been shown to produce parenchyma changes in the liver with granuloma formation [29], we measured liver enzymes such as alkaline phosphatase, lipase, AST, and ALT. There were no statistically significant changes among the experimental groups, suggesting that the liver function is not impaired (Table 1). Platelet aggregation was similar in both clopidogrel-dosed and clopidogrel-dosed/PG-PS-treated animals, indicating that PG-PS does not affect the generation of the active metabolite of clopidogrel (Figure 6). Thus, PG-PS has no apparent effect on liver function or clopidogrel metabolism to its active form.

The levels of the pro-inflammatory cytokines (IL-1, IFN-γ and IL-6) were much higher in the PG-PS-induced animals treated with clopidogrel compared to clopidogrel-treated animals alone. The increases in proinflammatory cytokines in the arthritic state are consistent with published studies [30], [31], and a further increase in these cytokines is correlated with the increase of inflammation observed by clopidogrel treatment. Patients afflicted with RA display a similar cytokine profile, including increased circulating levels of IL-1, IFN-γ and IL-6, similar to the increased levels we observed in the rat model with PG-PS in conjunction with clopidogrel [32]. Swennen *et al.*, [33], [34] reported an increase of IL-10 release upon stimulation of the P2Y12 receptor. Those findings may explain the dramatic decrease in IL-10 plasma levels observed in the PG-PS-induced arthritis animals treated with clopidogrel, and might thus explain why clopidogrel enhanced the inflammatory responses induced by PG-PS. This last effect indicates that clopidogrel might disrupt the natural cytokine cascade response as a result of an inflammatory insult induced by PG-PS, in which IL-10 becomes elevated in response to tumor necrosis factor (TNF)-α and IL-1.

Thrombocytosis was observed in the PG-PS+clopidogrel group but not in any of the other three groups during the chronic phase of inflammation. This finding might be related to platelet production by the bone marrow in response to inflammation. There was a trend towards a higher platelet count in the PG-PS group but this was not statistically different when compared to clopidogrel alone or with the untreated group. In contrast, we observed significant leukocytosis in the PG-PS and PG-PS+clopidogrel groups; in particular, the neutrophil count in the PG-PS group was significantly increased when compared to the clopidogrel and untreated groups. Thus, the leukocytosis observed in the PG-PS+clopidogrel group was due primarily to the addition of PG-PS. The differences between PG-PS and PG-PS+clopidogrel groups could be the result of certain metabolites of clopidogrel that may be acting on inflammatory cells as non-specific agonists. This last scenario may explain the increased levels of circulating pro-inflammatory cytokines characterizing the PG-PS+clopidogrel group. Whether the effects of clopidogrel on enhanced inflammatory responses are due to off-target effects of clopidogrel or its metabolites, or to a specific effect of the active metabolite on the platelet P2Y12 receptor remains to be established.

Previous studies using a K/BxN serum arthritis-induced murine model demonstrated the important role that platelet micro-particles play during the inflammatory changes observed in this animal model. The addition of clopidogrel increased ankle joint thickness and inflammation [19], in agreement with our findings; equally important, the clopidogrel side effect is observed in both mice and Lewis rats. Notably, clopidogrel has been of therapeutic benefit in other models used to study autoimmunity such as systemic lupus erythematosus where clopidogrel prevented kidney inflammation [35]. These last findings are not in discrepancy with our findings or those from Boilard *et al.* [19], since the pathophysiology of SLE is different from the one seen in RA.

Although our studies in rats cannot be directly extrapolated to human disease (RA), countless studies using the PG-PS rat model have been shown to correspond to human RA [14], [36], and thus this model is widely accepted in the scientific community for the study of the pathophysiology of RA. The fact that similar changes were observed in mice treated with clopidogrel [19] is supportive of our observations and it provides an additional experimental control tool (mouse model) to investigate the mechanism of action. Furthermore, although we used a dose of clopidogrel which is much higher than the dosage used clinically in humans, this strategy was carefully designed after previously standardized animal studies [37], [38] to enforce extensive arthritic responses. Thus, this model allowed us to investigate pronounced effects of PG-PS and clopidogrel in our rat models but these effects may be attenuated in clinical settings.

Observations from this study have translational clinical implications as the number of clinical reports in humans developing acute arthritis as a result of clopidogrel treatment is steadily rising [31], [39], [40], [41], [42], [43], [44]. These results point to potential concerns of clopidogrel treatment in patients with chronic arthritis, particularly rheumatoid arthritis, since those patients display a high cardiovascular risk and frequently require anti-thrombotic drugs including clopidogrel. Therefore, the data from our study strongly suggest that the use of clopidogrel should be used cautiously in patients with inflammatory diseases such as rheumatoid arthritis. In summary, this study utilizing the PG-PS-induced acute and chronic arthritis animal model provides compelling evidence for pro-inflammatory activity of clopidogrel treatment at least under the experimental conditions used. The pro-inflammatory effect of clopidogrel in the presence of PG-PS should be further evaluated to determine the mechanism(s) of action responsible for this detrimental effect.

## Materials and Methods

### Reagents

Purified, sterile PG-PS polymer from the cell walls of Group A, type 3, strain D58 *Streptococcus pyogenes* was obtained from BD Lee Laboratories (Grayson, GA). Clopidogrel was provided as 75 mg Plavix® tablets from Bristol-Myers Squibb/Sanofi Pharmaceutical partnership, New York, NY. A vehicle solution for clopidogrel, carboxyl methyl cellulose (CMC) and 2 MeSADP (2-methyl-thio-ADP) was purchased from Sigma-Aldrich Chemicals, St. Louis, MO. ELISA kits for IL-6 and IL-10 were purchased from R&D systems, Minneapolis, MN and IL-1β from Assay Designs Inc. Ann Arbor, MI. All other chemicals and reagents were purchased from Thermo Fisher Scientific Co, Waltham, MA.

Special equipment used in this study include: the Ultra-Call Mark III digital caliper to measure the ankle joint diameter (F.V. Flower Co. Inc., Newton, MA); the EZ 1500, anesthesia System from Euthanex Corp. (Palmer, PA) for isofluorane (VetOne Pharmaceuticals) to anesthetize the animals and the automatic Hemavet® Multispecies Hematology Systems (Drew Scientific, Inc. Oxford, CT) to count blood cells.

### Induction and Assessment of Arthritis

The Institutional Animal Care and Use Committee of Temple University School of Medicine approved this experimental protocol (approval number # ACUP3212). A total of 48 female pathogen-free Lewis rats (8-week old) weighing between 160–180 grams were used (Charles River Laboratories, Raleigh, NC). Animals were randomly separated into 4 different groups and studied for 5 days, acute phase; and 21 days, chronic phase. The untreated group received no treatment. The clopidogrel group received a 30 mg/kg daily oral dose of clopidogrel in 0.5% CMC (higher dose than the standard used in daily clinical practice in humans to magnify the therapeutic effect in animals; however, this dose has been used and standardized previously in animals) [37], [38]. The PG-PS group received a single dose of PG-PS 15 µg of rhamnose/gram of mean body weight, administered by *i.p.* injection on day 0, followed by oral administration of vehicle. The PG-PS+clopidogrel group received a single *i.p.* dose of PG-PS on day 0, followed by a daily oral dose of 30 mg/kg clopidogrel [37], [38]. Animals were weighed and examined daily. Under anesthesia, ankle diameter was measured and arthritis severity was assessed as previously described [29]. At times of sample collection (day 5 and 21), rats were anesthetized, and blood samples were collected by cardiac puncture for hematology, plasma separation and for serum collection for chemical analyses, using a 10∶1 ratio of blood in 3.8% sodium citrate as anticoagulant. Hematology studies were performed using the Hemavet® system. Serum samples were sent for chemistry analyses in Charles River Laboratories (Wilmington, MA). From each group, 4 animals were sacrificed on day 5 and 8 animals on day 21. After blood collection, animals were euthanized by cervical dislocation and tissue samples were collected.

### Plasma Preparations

Plasma was separated from blood cells by centrifugation in polypropylene tubes at 22°C at 100× g for 10 min. The platelet rich plasma was centrifuged at 22°C at 400× g for 10 min. Platelet-poor plasma was recovered and aliquots of supernatant were stored at −70°C for cytokine profile studies. Platelet pellets after centrifugation were resuspended in Tyrodes buffer (pH 7.4) containing 0.05 units/ml apyrase.

### Histopathology

Hind paws from all rats were collected, fixed in 4% buffered para-formaldehyde, decalcified in 5% formic acid and embedded in paraffin. Five micrometer sections were stained using hematoxylin and eosin for microscopic examination. A score system was utilized to determine the hind paw histopathological score. This system was used in this animal model by Espinola *et al.* [27] and was modified from the human scoring scale for rheumatoid arthritis patients made by Rooney and colleagues [45], [46]. For this purpose, eight pathological parameters were measured: synovium hyperplasia (# of cell depth), proliferative blood vessels (# of vessels per high power field (HPF)), sub-synovial fibrosis (percentage), inflammatory infiltrates (percentage of infiltrated vessels per HPF), intra-articular exudates, percentage of intra-articular space, cartilage erosion and subchondral inflammation. Each parameter received a severity value from 0 to 3. The histopathological score for each slide was the sum of the eight parameters (from 0 to 24) and the score means ± standard error from each group were plotted.

### Cytokine Profile

Plasma levels of IL-1β, IL-4, IL-6, IL-10, and IFN-γ were measured by the Luminex® System using rat-specific antibodies (Cytokine core laboratory of the University of Maryland School of Medicine). Plasma levels of IL-1β, IL-6, IL10 and PF4 were analyzed by ELISA, following the manufacturer's directions (PF4: Allied Biotech, Inc. Ijamsville, MD).

### Platelet Aggregation Studies

Aggregation of 0.5 mL washed platelets was analyzed using a P.I.C.A. Lumiaggregometer (Chrono-log Corp., Havertown, PA). Aggregation was measured using light transmission under stirring conditions (900 rpm) at 37°C. Each sample was allowed to aggregate for at least 3 min. The chart recorder (Kipp and Zonen, Bohemia, NY) was set for 0.2 mm/sec.

### Statistical Analysis

Data are presented as mean ± standard error of the mean (SEM) for each group. The joint diameter was analyzed as a continuous variable of all analyses. Statistical analysis of all the data was performed using one-way ANOVA; Bonferroni's Multiple Comparison Test was used as post-test analyses. *P*<0.05 was considered to be significant.
